# Can Compounds of Natural Origin Be Important in Chemoprevention? Anticancer Properties of Quercetin, Resveratrol, and Curcumin—A Comprehensive Review

**DOI:** 10.3390/ijms25084505

**Published:** 2024-04-19

**Authors:** Elżbieta Cecerska-Heryć, Zofia Wiśniewska, Natalia Serwin, Aleksandra Polikowska, Małgorzata Goszka, Weronika Engwert, Jaśmina Michałów, Maja Pękała, Marta Budkowska, Anna Michalczyk, Barbara Dołęgowska

**Affiliations:** 1 Department of Laboratory Medicine, Pomeranian Medical University of Szczecin, Powstancow Wielkopolskich 72, 70-111 Szczecin, Poland; 67994@student.pum.edu.pl (Z.W.); natalia.serwin@pum.edu.pl (N.S.); polikowska.aleksandra@gmail.com (A.P.); malgosia@goszka.pl (M.G.); 83688@student.pum.edu.pl (W.E.); 83663@student.pum.edu.pl (J.M.); majapekala0@gmail.com (M.P.); barbara.dolegowska@pum.edu.pl (B.D.); 2 Department of Medical Analytics, Pomeranian Medical University of Szczecin, Powstancow Wielkopolskich 72, 70-111 Szczecin, Poland; marta.budkowska@pum.edu.pl; 3 Department of Psychiatry, Pomeranian Medical University of Szczecin, Broniewskiego 26, 71-460 Szczecin, Poland; anna.michalczyk@pum.edu.pl

**Keywords:** resveratrol, quercetin, curcumin, chemoprevention, oxidative stress, antioxidants

## Abstract

Malignant tumors are the second most common cause of death worldwide. More attention is being paid to the link between the body’s impaired oxidoreductive balance and cancer incidence. Much attention is being paid to polyphenols derived from plants, as one of their properties is an antioxidant character: the ability to eliminate reactive oxygen and nitrogen species, chelate specific metal ions, modulate signaling pathways affecting inflammation, and raise the level and activity of antioxidant enzymes while lowering those with oxidative effects. The following three compounds, resveratrol, quercetin, and curcumin, are polyphenols modulating multiple molecular targets, or increasing pro-apoptotic protein expression levels and decreasing anti-apoptotic protein expression levels. Experiments conducted in vitro and in vivo on animals and humans suggest using them as chemopreventive agents based on antioxidant properties. The advantage of these natural polyphenols is low toxicity and weak adverse effects at higher doses. However, the compounds discussed are characterized by low bioavailability and solubility, which may make achieving the blood concentrations needed for the desired effect challenging. The solution may lie in derivatives of naturally occurring polyphenols subjected to structural modifications that enhance their beneficial effects or work on implementing new ways of delivering antioxidants that improve their solubility and bioavailability.

## 1. Introduction

Globally, cancer is the second leading cause of death, accounting for one in six deaths. In 2018, approximately 9.6 million people died of cancer worldwide, and an increasing trend in the incidence is currently observed [1]. Based on this information, increasing attention is being paid to developing methods to prevent cancer through chemoprevention. Currently, it is increasingly suggested that there is a relationship between the occurrence of cancer and redox imbalance in the body [2]. Neoplastic transformation is induced by, among others, inflammation, cytokines, oncogenes, intensive metabolism associated with continuous proliferation, mutations in mitochondrial DNA, and dysfunctions in the respiratory chain. This produces excessive amounts of reactive oxygen and nitrogen species, which are molecules that damage proteins, lipids, and DNA [3]. High concentrations of ROS in cancer cells may lead to cellular adaptation, an increase in the rate of proliferation, the formation of mutations in DNA, and consequently, genome instability, as well as resistance to specific groups of drugs used in chemotherapy. One of the potential methods of preventing cancer is using natural antioxidants, which, thanks to their properties, help restore the body’s homeostasis. Such substances include polyphenols of plant origin, which have a wide range of health applications. The results of epidemiological studies indicate that a long-term, regular diet rich in these compounds may prevent cancer development [2]. This comprehensive review aims to evaluate the impact of the antioxidant properties of three natural polyphenols—resveratrol, curcumin, and quercetin—on cancer prevention through mechanisms that reduce oxidative stress and possibly support anticancer therapy. It also aims to assess the advantages and disadvantages of use, problems with bioavailability and absorption of these polyphenols, and current knowledge on the subject.

## 2. Oxygen, Oxidative and Nitrosative Stress

Aerobic organisms are energetically benefitted by oxidants, which help their survival during their metabolic processes while also being a cause of oxidative stress. The lungs are especially exposed to oxygen in humans, as about 5% of exhaled O_2_ is converted to reactive forms [4,5].

Reactive oxygen species (ROS) are formed due to the presence of O_2_ in the cells and include free radicals and non-radical compounds. Oxygen in atmospheric air exists in its primary state, taking the triplet form, which is characterized by the lowest reactivity [6,7]. For a reaction of triplet oxygen with another molecule to be possible, the reactant would also have to be in triplet form. Meanwhile, a large proportion of organic compounds present in the body take a single form in the primary state, where the electrons are paired. For this reason, it is not easy for oxygen to encounter a molecule with which a reaction involving two electrons could occur. Instead, there is a possibility of O_2_ taking a single electron from the reactant, so free radical formation occurs [6].

Triplet oxygen can be transformed into forms characterized by higher reactivity by excitation, such as UV radiation or some chemical reactions. As a result of energy transfer, the oxygen molecule transforms from a triplet form into a singlet form, characterized by higher reactivity and better oxidizing properties [7,8]. Electron reactions, on the other hand, produce reactive oxygen species such as O •−, H O, and •OH [6].

Oxidative stress can be described as a state of disturbed balance between oxidative mechanisms associated with producing reactive oxygen species and antioxidant mechanisms that play a defensive role in the body [9]. Among ROS, oxygen free radicals and non-radical forms can be distinguished. The first group mentioned includes superoxide anion radicals and more reactive individuals such as hydroxyl, alkoxy, alkyl, peroxyl, and peroxy radicals. In contrast, the second group comprises hydrogen peroxide, ozone, singlet oxygen, hypochlorous acid, and hypobromous acid [3,6,10]. Reactive oxygen species belonging to the free radical group are characterized by having a minimum of one unpaired electron on their valence shell. They seek to lose this electron or gain another one from another molecule to achieve pairing, making them highly reactive as a result. They thus cause the formation of new free radicals while achieving stability themselves [11]. As a result of the reaction of ROS with organic molecules, free radicals of organic substances occur [6].

While exploring the concept of oxidative stress, it is worth mentioning that it is closely related to the matter of nitrosative stress, during which an increase in the concentration of reactive nitrogen species (RNS) occurs. A concise duration in the body and an ability to affect cells physiologically and in pathological processes are standard characteristics of reactive oxygen and nitrogen species [11,12]. Nitric oxide can react with superoxide anion radicals, leading to reactive oxygen species and peroxynitrite formation. In addition to the molecules mentioned, the other RNS are nitrogen dioxide, nitrile cation, nitrosyl cation, and nitrosyl anion [3,13].

## 3. Sources of Reactive Oxygen and Nitrogen Species

**Figure 1:**
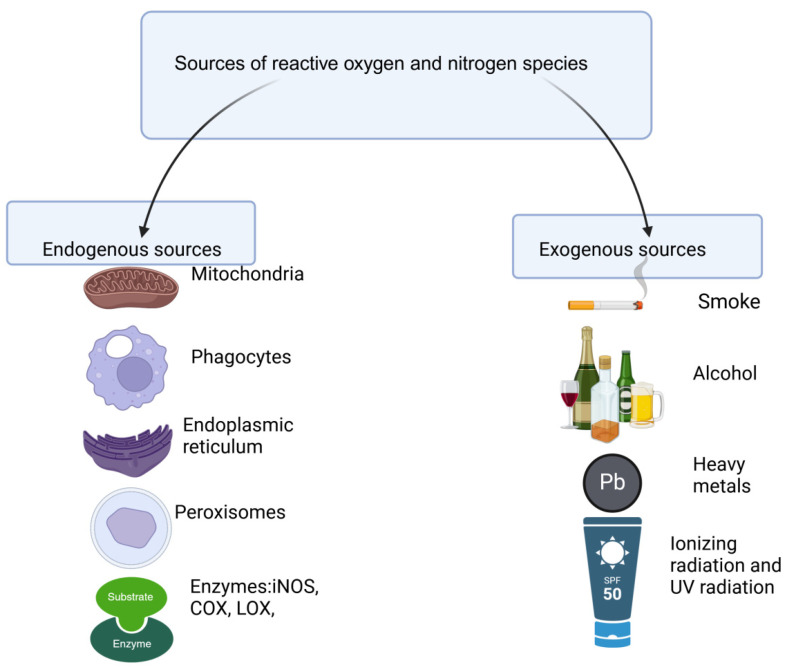
**Endogenous and exogenous sources of reactive oxygen and nitrogen species.** The figure shows endogenous (mitochondria, phagocytes, endoplasmic reticulum, peroxisomes, and iNOS, COX, LOX) and exogenous (smoke, alcohol, heavy metals, and radiation) sources of reactive oxygen species [14,15,16,17,18,19,20,21,22,23,24]. It was created by BioRender https://app.biorender.com/ (accessed on 25 March 2024).

Whether reactive oxygen and nitrogen species arise from reactions originating in the body or their presence is due to the surrounding environment, they can be distinguished analogously between their endogenous and exogenous sources (Figure 1). ROS and RNS formed with the participation of mitochondria, phagocytes, endoplasmic reticulum, peroxisomes, inducible nitric oxide synthase (iNOS), xanthine oxidase (XO), lipoxygenase (LOX), cyclooxygenase (COX), or in the process of autoxidation of small molecules and hemoglobin, have endogenous origin [14,15,16]. Mitochondria play a significant role, as it is estimated that they can produce up to 90% of ROS formed in the body [14]. In these organelles, the sequence of reactions results in the reduction of O_2_ to water and the production of adenosine triphosphate. For the reduction to be complete, there must be four transported electrons [17,18]. Some of the transported electrons, however, leave the enzyme complex assembly. About 1–4% of O_2_ in the mitochondria is estimated to undergo single-electron reduction following superoxide anion radical formation [14]. Subsequent reactions result in the attachment of single electrons and produce hydrogen peroxide, a hydroxyl radical, and two water molecules [19]. The mitochondrial respiratory chain, under hypoxic conditions, can also become a source of nitric oxide, from which other RNS are formed during further transformations.

Exogenous sources, on the other hand, include exposure to tobacco smoke, alcohol consumption, pollutants found in the environment, heavy metals, and UV and ionizing radiation [20,21,22]. Reactive forms are also created due to the oxidation of xenobiotics, such as drugs or poisons [23]. Another factor relevant to the amount of ROS and RNS formed in the body is diet because the foods consumed may have varying levels of antioxidants [21] (Figure 1). These compounds can prevent oxidation of the body’s molecules, thereby protecting them from adverse changes in function and structure [24]. Therefore, a diet low in antioxidants will be associated with forming more reactive species [21].

## 4. Carcinogenesis

Carcinogenesis is a series of changes in the body that result in cancer development. This complex process is often divided into three main phases: initiation, promotion, and progression. The initiation phase is associated with the occurrence of mutations in DNA, the transformation of protooncogenes into oncogenes, and the inactivation of suppressor genes, as a result of which the control of cell proliferation and differentiation is disrupted [22]. Under normal conditions, protooncogenes regulate processes under the control of transcription and mitogenic (mitosis-inducing) factors and participate in the transduction of intercellular signals. At the same time, the final product of oncogene expression may be a protein with excessive enzymatic activity, or increased production of this protein. In addition, a loss of regulatory mechanisms takes place. Suppressor genes inhibit proliferation and induce apoptosis so that there is an average number of cells without accumulated damage.

In contrast, inactivation of any of these genes leads to loss of cell cycle control [23]. Exposure of cells to carcinogens such as ultraviolet and ionizing radiation, oncogenic viruses, or aromatic hydrocarbons in cigarette smoke can contribute to the initiation stage. Mutations are likely due to DNA polymerase error and spontaneous initiation [24]. If carcinogenesis proceeds, clonal growth of the cell occurs at the second level. Both genetic and epigenetic changes stimulate the proliferation process [22]. Factors called promoters influence the acceleration of tumor development during the promotion phase. The changes caused by them are reversible, in contrast to those induced during initiation. Eventually, at the progression stage, the degree of instability of the karyotype increases, and the ability to metastasize and invade is developed. However, several subsequent mechanisms, such as angiogenesis, cell migration, and evasion of the body’s immune response, are needed [22,25,26].

## 5. Reactive Oxygen Species in Carcinogenesis

**Figure 2:**
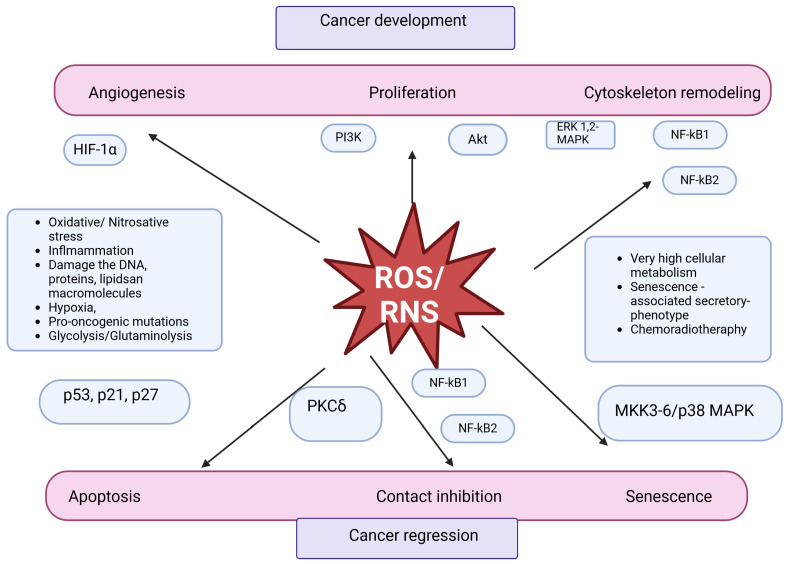
**Role of reactive and nitrogen oxygen species in carcinogenesis.** The double-faced ROS/RNS in cancer. High levels of ROS/RNS, resulting from abnormal cellular metabolism and inflammation, promote tumor proliferation, vascularization, and metastasis. At the same time, an excessive amount of ROS/RNS is likely to induce senescence and/or apoptosis [26,34].

Among the properties of agents classified as carcinogens is the induction of ROS formation, which leads to chain reactions that generate further reactive species [27]. Chronic infections, asbestos, radiation, autoimmune diseases, and many other stimuli can contribute to chronic inflammation. As a result, at the site of exposure to a given agent, activation of phagocytes, an oxygen explosion (sudden release of large amounts of ROS by macrophages and leukocytes), and the release of pro-inflammatory cytokines occur. During this state, the production of reactive oxygen and then nitrogen species increases, which can damage the genetic material of cells and lead to the initiation of carcinogenesis [28]. Cells also contain NADPH oxidases, NOX (NADPH oxidase), and DUOX (dual oxidases), which specifically produce ROS in many different tissues. Although NOX/DUOX may be involved in maintaining optimal redox levels in cells, there is also accumulating evidence that ROS from NADPH oxidases may increase the risk of genome instability and consequently cause cancer. Cancer cells can produce large amounts of ROS, and their source may be the deregulation of NOX/DUOX, as in the case of prostate cancer (NOX1 and NOX5), melanoma, and glioblastoma multiforme (NOX4), among others. Recent studies have shown that targeting NADPH oxidases with NOX inhibitors can impair tumor growth in vivo, indicating that these proteins may be helpful to targets in future clinical strategies against cancer [29]. Chronic inflammation also has adverse effects at later stages, associated with promotion, angiogenesis intensity, proliferation, or the ability to metastasize [20]. Oxidative stress causes the activation of inflammatory pathways, especially those involving nuclear factor kappa B (NF-κB) [27]. Reactive oxygen species damage DNA, lipids, proteins, and macromolecules, and proliferating cells resist apoptosis. Eventually, they can invade and metastasize, and angiogenesis progresses [26]. The order of changes occurring in the body under the influence of reactive oxygen species that affect carcinogenesis is shown in Figure 2.

In cancer cells, the production of reactive oxygen species is usually increased. Cell metabolism remains high, and the hypoxic state increases ROS production. These molecules, as well as the subsequently formed oxygen and nitrogen derivatives, are involved in cell signaling transduction, which affects the degree of survival, tumor progression, and resistance to the applied therapy [30]. Nevertheless, sufficiently high concentrations of ROS and RNS can contribute to the cure of the disease. Much attention is paid to NO regarding the effects of reactive nitrogen species on cancer cells [31]. However, cancer develops mechanisms that allow it to survive under increased production of reactive oxygen species by maintaining their concentrations below those that would lead to a cytotoxic effect. This can happen, among other things, due to the production of antioxidant enzymes when increased expression of transcription factors involved in regulating redox homeostasis occurs. Thus, the amount of ROS is still maintained at a level that has a pro-cancer effect. Treatment methods, including radiotherapy and chemotherapy, induce the accumulation of reactive oxygen species and lead to exceeding the cytotoxicity threshold [32,33].

## 6. Nitrogen Species in Carcinogenesis

There are many reports in the literature on the relationship between chronic inflammation, increased risk of cancer, and increased levels of nitric oxide (NO) [35]. Tissue damage is manifested by the production of reactive nitrogen species (RNS), such as peroxynitrite (ONOO−), nitrogen dioxide (•NO_2_), and nitrous anhydride (N_2_O_3_) [36,37]. RNS can directly deaminate DNA bases (4,5). Exocyclic DNA adducts such as 1, N6-ethenoadenine (εA), and N2,3-thioguanine can also react with DNA via secondary lipid peroxidation products [38,39]. Several DNA glycosylases of the base excision repair (BER) pathway initiate the repair of RNS-induced and oxidative DNA base adducts [40]. Alkyladenine DNA glycosylase (AAG) appears to be the major mammalian DNA glycosylase, removing hypoxanthine, εA, and N2,3-ethenoguanin [41,42,43,44,45,46]. Several studies have shown that levels of the AAG substrate, εA, increased in chronic inflammatory conditions in murine models [47]. Additionally, elevated ethene adducts have been observed in colonic polyps from patients with familial adenoma [48]. RNS can also modify proteins through nitration and nitrosation, two types of post-translational modifications that can regulate protein function and alter signaling pathways [49,50,51,52]. Two other BER components, the DNA glycosylase OGG1 and APE1, are post-translationally modified by RNS [53,54]. Protein exposure to nitrating RNS, OONO−, and •NO_2_ may form nitraducts on aromatic groups such as tyrosyl residues. Nitrosating RNS such as N_2_O_3_ attack nucleophilic sites, especially cysteinyl thiolates. In this regard, AAG represents an intriguing target due to the number of tyrosine and cysteine residues near the active site. A specific modification of the RNS may differentially modulate AAG activity. The demonstration of nitrated AAG in colon cancer patients suggests that this RNS modification may be an important factor contributing to BER imbalance in adenocarcinoma [55].

NO also has a vast meaning in carcinogenesis in solid tumors. For example, NO has been described as very important in metabolic crosstalk between cancer cells and other members of the cancer microenvironment. NO can promote tumor aggressiveness and the development of a chemotherapy-resistant phenotype by simulating hypoxic conditions (even in the presence of oxygen), promoting glycolysis, and diminishing mitochondrial respiration. NO-related intracellular hypoxia provokes tumor progression through multiple pathways, including increasing the glycolytic process and augmented consumption of glutamate in the TCA cycle [56]. Although NO can mediate the development of chemotherapeutic resistance, extensive data confirm its potential to re-establish malignant cell sensitivity to immune-cell-mediated antitumor activities and make tumors sensitive to chemotherapy [57]. ROS/RNS can also mediate mechanisms of targeted cancer therapy. The modulation of the ROS/RNS balance increases the oxidative stress to a level that overwhelms the antioxidant capacity of cancer cells. Moreover, these treatments influence reactive oxygen and nitrogen species, which function as second messengers and, in this way, affect redox signaling. Such agents are designed to target VEGFR, EGFR, HER, BRAF, and PDGFR. Still, in contrast to beneficial effects, some of these agents might increase the antioxidant capacity of cancer cells by upregulating GSH levels, contributing to cancer cell growth and survival. In the future, special attention should be given to the possibility that oxidative and nitrosative stress biomarkers might predict the therapeutic response [56].

## 7. Chemoprevention

Some synthetic and natural substances have been proven to help stop or reverse carcinogenesis, and their use for this purpose is called chemoprevention. Certain drugs and compounds in plants possess such properties; they can be called chemopreventive agents [58]. Cancer chemoprevention is a significant cancer preventive strategy that utilizes naturally occurring dietary phytochemicals or therapeutic drugs with relatively low toxicity. Phytochemicals, along with physical activity and mental relaxation, can inhibit, retard, or reverse carcinogenesis. With modern technology and instrumentation, many studies on dietary phytochemicals have been performed (including their chemistry, biological activities, and mechanisms of action at the cellular level, in in vivo animal model systems, and in clinical trials). Carcinogenic species, such as environmental pollutants, dietary mutagens, and radiation, may produce ROS and/or reactive nitrogen species (RNS), which react with cellular molecules such as proteins, lipids, and DNA to induce carcinogenesis [59].

There are different strategies to stop carcinogenesis. Blocking agents act at the initiation stage, and suppressive agents show activity at two levels: during promotion and progression. The first group of compounds can alter the metabolism of the carcinogen, neutralize ROS/RNS and reactive metabolites, and cause an increase in repair and detoxification. In situations where the initiation phase has already occurred, suppressive agents promote apoptosis, reduce cell proliferation, eliminate reactive oxygen species, inhibit inflammation, and alter gene expression [58]. However, many compounds show chemopreventive properties at all three stages of carcinogenesis. Examples include the following antioxidants: curcumin, resveratrol, and quercetin [60,61,62]. Taking chemopreventive measures is recommended for healthy individuals with a high risk of cancer (genetic burden, chronic exposure to carcinogens), patients with a known precancerous condition, and those cured but who appear at risk of recurrence. Another option available is using chemopreventive agents as a method of supporting chemotherapy [58].

### Role of Antioxidants in Chemoprevention

**Figure 3:**
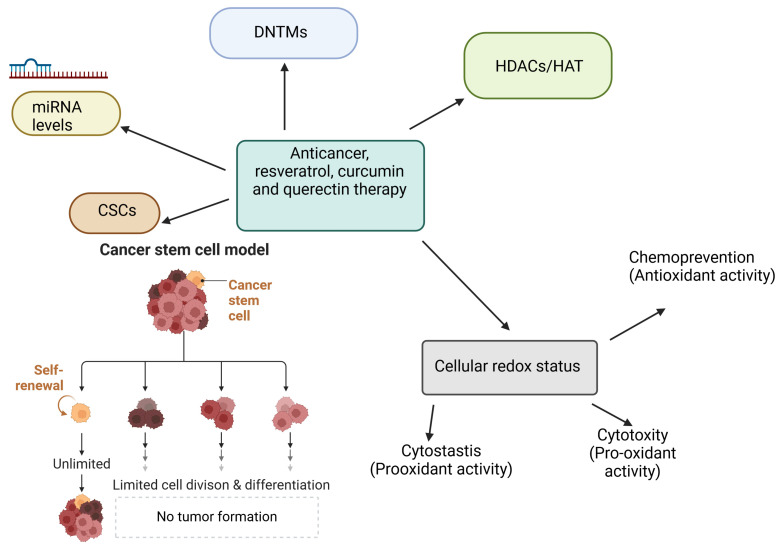
**Targets of polyphenol anticancer therapies.** Epigenetic pathways, cellular redox status, and cancer stem cells are therapeutic targets for resveratrol, curcumin, and quercetin anticancer therapies. Pro-oxidant activity may induce high ROS-mediated cytotoxicity or low ROS-mediated cytostasis depending on acute or chronic treatment. According to the figure, several natural compounds induce a pro-oxidant apoptotic mechanism at high concentrations, whereas low doses and chronic exposure trigger ROS-epigenetic mediated cellular senescence. DNMTs—DNA methyl transferases; HDACs/HATs—histone deacetylases/histone acetyltransferases; CSCs—cancer stem cells; miRNAs—microRNAs [66].

Recently, authors of scientific publications have pointed to antioxidants as antitumor agents due to their ability to reduce oxidative stress levels, which means diminished DNA damage [60]. On the one hand, oxidative stress is a factor in the development of tumorigenesis. Still, on the other hand, high levels of ROS/RNS can also have an anticarcinogenic effect, as is the case with radiation therapy or chemotherapy. Moreover, antioxidants can also manifest pro-oxidant properties under certain conditions, so their use in chemoprevention is not limited to reducing reactive oxygen or nitrogen species. Depending on the concentration and duration of cell exposure to a given compound, the production of ROS/RNS can be induced until their levels increase enough to damage and kill cancer cells [58,59]. In the case of several antioxidants with potential applications against carcinogenesis, their selectivity has been demonstrated to destroy cancerous cells without the observed adverse effects on normal ones, also through mechanisms without the involvement of reactive oxygen and nitrogen species [63,64,65]. Dietary phytochemicals directly scavenge ROS/RNS and indirectly remove carcinogenic reactive intermediates via the transcription factor Nrf2 (nuclear factor erythroid 2 p45 (NF-E2)-related factor 2) antioxidant and detoxification system. When Nrf2 is released from Kelch-like ECH-associated protein 1 (Keap1) and translocates to the nucleus, Nrf2 binds to antioxidant-responsive elements (AREs) in the promoter/enhancer region of phase II detoxification and antioxidant enzyme genes with the Maf (Musculoaponeurotic fibrosarcoma) protein [58,59]. Given the facts presented, the chemopreventive properties of antioxidants can be explained by the existence of various ways to prevent carcinogenesis. At the same time, the main topic of this article remains the antioxidant nature of selected compounds and their participation in this complex process (Figure 3).

## 8. Resveratrol

One of the antioxidants of interest to modern science is resveratrol (3,5,4′-trihydroxystilbene). This substance belongs to polyphenols, which are compounds produced by plants, and the highest concentrations can be found in blueberries, blackberries, mulberry fruit, and red grapes. Among grapevines such as Vitis vinifera, Vitis labrusca, and Vitis rotundifolia, the resveratrol content in the skins and seeds can range between 50 and 100 μg/g [67]. Valuable sources of the described compound are also knotweed root, peanuts, and beverages such as wine and tea. This polyphenol is produced by more than 70 plant species, including some trees, such as spruce and eucalyptus [68,69,70]. 3,5,4′-trihydroxystilbene can also be classified as a phytoalexin, as this group of compounds is produced by plants under stress conditions [71]. 

The synthesis of resveratrol occurs due to fungal infections and is intended to protect against the pathogen [72]. In addition, its production occurs due to mechanical trauma or UV radiation. The synthesis of this compound is mediated by stilbene synthase from a single molecule of p-coumaroyl-coenzyme A [67]. Resveratrol exists naturally in two isoforms: trans- and cis-isomer. Trans-resveratrol is the more stable of the two forms, and its physiological effect has been better understood than cis-resveratrol. Although trans-resveratrol is the more active isomer in anticancer and antioxidant activity, the other form also possesses such properties [73,74].

### 8.1. Effect of Resveratrol on Metabolism and Detoxification of Carcinogens

**Figure 4:**
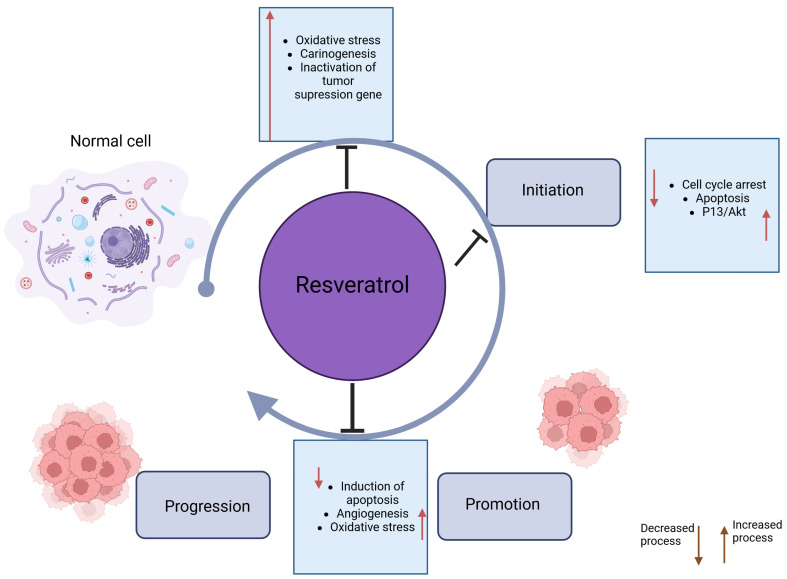
**The importance of resveratrol in carcinogenesis**. Resveratrol plays a role in cancer management by inhibiting the initiation, promotion, and progression of carcinogenesis and modulating key cell signaling molecules [80].

Resveratrol’s chemopreventive effects result in large part from its influence on mechanisms related to carcinogen metabolism, which include modification of the action of antioxidant enzymes and those contributing to the generation of ROS and RNS, lipid peroxidation, and even signaling pathways related to the development of inflammation. The antioxidant properties of 3,5,4′-trihydroxystilbene associated with chemoprevention may have a different impact and final effect depending on the stage of carcinogenesis. In the case of the first phase, initiation, they may relate to inhibiting the metabolic activation of carcinogens and carrying out their detoxification. This process is made possible by affecting the enzymes involved in the phase I reaction, cytochrome P450 (CYP) enzymes, where the carcinogen would be activated without the presence of the antioxidant, and phase II, during which enhanced detoxification occurs after modification by the antioxidant. In addition, CYP enzymes contribute to some extent to the intracellular production of reactive oxygen species and the formation of oxidative damage. Resveratrol has been shown to stop the activity of CYP1A1, 1A2, and 1B1 enzymes. Recent publications indicate that the 1B1 enzyme is also linked to cancer promotion and progression [75]. Administration of resveratrol to mice suppressed expression of the CYP1A1 enzyme and prevented the formation of DNA adducts due to the animals’ exposure to benzo[a]pyrene and the action of oxidative stress occurring under its influence [76,77]. Under the influence of resveratrol, the expression and activity of CYP1B1 induced by the administration of 2,3,7,8-tetrachlorodibenzo-p-dioxin (TCDD) were inhibited, so the production of catechol estrogens and, consequently, their oxidation did not occur either. This prevented DNA damage and possibly neoplastic transformation of MCF-10A human mammary epithelial cells, that is, the appearance of cancer cells [78]. In studies, the compound also showed the induction of phase II detoxification enzymes such as glutathione S-transferase (GST), glucuronyltransferase, and NAD(P)H: quinone reductase [77]. Additionally, in one study in mice whose skin was exposed to 12-O-tetradecanoylphorbol-13-acetate (TPA), prior administration of resveratrol restored glutathione concentrations and conferred superoxide dismutase activity to levels comparable to controls [79] (Figure 4).

### 8.2. Resveratrol Eliminates ROS and Prevents DNA Synthesis and Its Oxidative Damage

Resveratrol reduces oxidative stress in vivo and in vitro. In a B16 mouse melanoma cell line, reactive oxygen species production and proliferation were halted in the presence of this antioxidant [81]. In another experiment, human HL-60 leukemia cells were exposed to TPA, and resveratrol inhibited the formation of free radicals associated with this exposure [82]. In a rat experiment, administering this polyphenol first and then the carcinogen potassium bromate (KBrO3) prevented oxidative DNA damage in the animals’ kidneys [83]. In another study, the same antioxidant alleviated oxidative stress induced in rat pheochromocytoma cells (PC12 cell line) by iron ions and t-butyl hydroperoxide (tBHP). It was shown that combining resveratrol with vitamin C or E produced a more significant protective effect than using these substances separately [84]. In one experiment, 3,5,4′-trihydroxystilbene reduced the severity of oxidative stress during carcinogenesis induced in rat liver by exposure to diethylnitrosamine (DENA) [85]. This polyphenol can also counteract the increase in reactive oxygen species after exposure of cells to oxidizing agents such as hydrogen peroxide, UV radiation, and tobacco smoke condensate [60]. In two different independent experiments, the application of 3,5,4′-trihydroxystilbene in the samples was followed by a reduction in H_2_O_2_-induced oxidative DNA damage in breast cancer cells and an arrest in the accumulation of ROS and DNA strand breaks in cells under the influence of tobacco smoke condensate [86,87]. It has also been shown that resveratrol can partially counteract tumor progression. As reported by Fontecave et al. [88], this antioxidant in a mouse lymphoblastic leukemia cell line caused inhibition of DNA polymerase and deoxyribonucleotide synthesis by eliminating the tyrosyl radical formed by ribonucleotide reductase, thus stopping the growth of tumor cell lines.

### 8.3. Resveratrol and Regulation of Nitric Oxide Synthesis

Holian et al. [89] demonstrated that resveratrol stimulated nitric oxide synthase activity in the SNU-1 human gastric adenocarcinoma cell line to produce lower levels of NO and thus exerted antioxidant effects. Cancer cells first treated with low concentrations of hydrogen peroxide responded by increasing DNA synthesis and proliferation. Administration of resveratrol reversed this effect. Based on the collected data, it was suggested that at the concentrations of nitric oxide generated by the presence of resveratrol, NO molecules reacted with endogenously produced reactive oxygen species, influenced the inhibition of proliferation of SNU-1 cells, and could potentially be the source of induction of the apoptosis process. In an experiment conducted by Panaro et al. [90], resveratrol decreased the expression of inducible nitric oxide synthase and inhibited nitric oxide production. The experiment was performed on the human colorectal adenocarcinoma cell lines Caco-2 and SW480, which were previously exposed to lipopolysaccharide. Similar results were obtained by conducting studies on the DU145 prostate cancer cell line, where resveratrol stopped its growth while reducing NO levels and inhibiting iNOS [82].

### 8.4. Effect of Resveratrol on Lipid Metabolism

Due to its antioxidant properties, resveratrol counteracts lipid peroxidation in cell membranes by capturing the radicals produced [64]. This polyphenol significantly reduces the copper-mediated oxidation of low-density lipoproteins (oxidized LDL, oxLDL). Oxidized LDL is not only involved in the development of atherosclerosis—from the point of view of oncology, it is essential to contribute to increased survival of colon, prostate, and breast cancer cells [90]. Indeed, elevated levels of oxLDL and their receptors have been linked to an increased risk of various types of cancer. Oxidative modification of lipoproteins can damage DNA and have mutagenic effects, leading to carcinogenesis [91].

In an experiment in which carcinogenesis was initiated in Sprague–Dawley rats through the use of 7,12-dimethylbenz(α)anthracene (DMBA), the efficacy of resveratrol as an inhibitor of 5-lipoxygenase (5-LOX), an enzyme that affects the development and progression of human cancers and which becomes overexpressed in such a situation, was tested. The generation of superoxide anion radicals also occurs during the reactions catalyzed by 5-LOX in a physiological state. In addition, the relationship between the intensity of lipid peroxidation, DNA damage, and antioxidant supplementation was studied. The polyphenol tested appeared to act as a 5-lipoxygenase inhibitor, reduce lipid peroxidation, and prevent oxidative damage to DNA [92].

Through its antioxidant properties, resveratrol also counteracted UVB-induced damage to human keratinocytes, which can cause skin cancer. Moreover, the polyphenol blocked the NF-κb pathway associated with inflammation and carcinogenesis activated by UVB. The antioxidant’s reduction of oxidative stress inhibited this pathway [93].

### 8.5. Effect of Antioxidant Properties of Resveratrol on Cyclooxygenases 

In one study, Martinez and Moreno [94] reported that resveratrol inhibited the formation of superoxide anion radical and hydrogen peroxide produced by macrophages stimulated with PMA or lipopolysaccharides (LPS). Also, a reduction in arachidonic acid release and attenuation of cyclooxygenase-2 (COX-2) induction occurred. These phenomena were initiated by PMA, LPS, O•−, and HO. They were also associated with reduced prostaglandin synthesis. Given the results obtained in this experiment, the impact of the antioxidant action of resveratrol on the degree of arachidonic acid release and COX-2 induction was suggested. Overexpression of the mentioned enzyme is found in many types of cancer, such as gastric, colorectal, pancreatic, lung, esophageal, breast, prostate, bladder, endometrial, head, and neck cancers. In addition, COX-2 is involved in generating and maintaining inflammation [95]. Thus, due to its association with carcinogenesis, COX-2 is currently the focus of research, including interactions with antioxidants such as resveratrol.

Feng et al., in a study conducted on colon cancer cells, showed that colon cancer cells exposed to resveratrol showed significantly lower cyclooxygenase-2 and prostaglandin receptor expression. Treatment of colon cancer cells with the inhibitor of cyclooxygenase-2, indomethacin, and administration of silencer RNA for cyclooxygenase-2 also produced similar results [96].

In turn, Zheng et al., in a study conducted on rats, showed that the administration of resveratrol, both intragastric (IG) injection and intraperitoneal (IP), in two-day intervals for 30 weeks would be of value in thyroid tumor prevention [97].

Harikumar et al. studied mice suffering from cancer (PaCa). The study aimed to sensitize PaCa to gemcitabine in vitro and in vivo. They established PaCa xenografts in nude mice, randomly divided into four groups, and treated with vehicle, gemcitabine, resveratrol, and the combination. NF-κB modulation and markers of proliferation, angiogenesis, and invasion were determined by electrophoretic mobility shift assay (EMSA), immunohistochemistry, and Western blot analysis. Resveratrol inhibited the proliferation of four different human PaCa cell lines, synergized the apoptotic effect of gemcitabine, and inhibited the constitutive activation of NF-κB and the expression of bcl-2, bcl-xL, COX-2, cyclin D1, MMP-9, and VEGF. They found that resveratrol significantly inhibited tumor growth (*p* < 0.001), and this effect was further enhanced by gemcitabine (*p* < 0.001). Both the Ki-67 proliferation index markers and CD31 microvessel density were significantly reduced in tumor tissue by combining gemcitabine and resveratrol (*p* < 0.001 vs. control; *p* < 0.01 vs. gemcitabine). Compared with vehicle control, resveratrol inhibited NF-κB activation and the expression of cyclin D1, COX-2, ICAM-1, MMP-9, and survivin. The results show that resveratrol can enhance the effects of gemcitabine by suppressing markers of proliferation, invasion, angiogenesis, and metastasis [98].

Another known enzyme of the same family is cyclooxygenase-1 (COX-1), which contributes to the generation of ROS in the body. Resveratrol’s antioxidant properties involve reducing its activity. The inhibitory effect of 3,5,4′-trihydroxystilbene has been confirmed in various studies, including experiments conducted on rat microglia whose cells were treated with LPS. As with cyclooxygenase-2, COX-1 expression also increases in numerous cancers, and in some of them, such as serous ovarian cancer, it plays a key role [99].

### 8.6. Interaction between Resveratrol and Nrf2

The results of one experiment conducted in recent years indicate that resveratrol has a protective effect through a signaling pathway mechanism involving Nrf2 (nuclear factor erythroid 2-related factor 2). Among the functions of this molecule is the regulation of genes related to the coding of antioxidant enzymes such as heme oxygenase, glutathione S-transferase, and catalase, which makes it an essential element in terms of maintaining redox homeostasis and the cellular response to oxidative stress [83,100]. 3,5,4′-trihydroxystilbene prevented damage in porcine enterocyte IPEC-J2 cells induced by deoxynivalenol, which is a substance that causes the production of reactive oxygen species and an important factor in carcinogenesis. The Nrf2 signaling pathway has been suggested as a mechanism for inhibiting intracellular oxidative stress, i.e., the antioxidant effect of resveratrol. These conclusions were made based on assessing the transcripts of genes affecting antioxidant defense, such as SOD1 (copper–zinc superoxide dismutase), GCLC (glutamate cysteine ligase catalytic subunit), and GCLM (glutamate cysteine ligase modulatory subunit), and measuring the concentration of Nrf2 itself in the cell nucleus. The polyphenol tested caused both an increase in the expression of all three genes and increased nuclear accumulation of the transcription factor [83]. An experiment conducted by Rubiolo et al. [101] provided similar results. Rat liver cells were exposed to oxidative stress by adding t-butyl hydroperoxide to the medium. Cells then exposed to resveratrol were characterized by higher levels of Nrf2 factor than controls. Accordingly, an increase in the concentrations of enzymes involved in phase II detoxification (SOD, GPx, CAT, GR, GST, and NAD(P)H: quinone reductase) was also observed, and it has been suggested that cellular resistance to oxidative stress was improved through this mechanism. Kang et al. [102] demonstrated a reduction in the accumulation of reactive oxygen species and DNA damage in mammary epithelial cells with an inactive BRCA1 gene, a suppressor strongly associated with breast cancer development, through resveratrol. The concomitant increase in Nrf2 activity may indicate that this factor protects against oxidative stress.

### 8.7. Link between Resveratrol and Tumor Necrosis Factor

TNF (tumor necrosis factor) belongs to the group of cytokines. It is involved in physiological and pathological processes and pro- and anti-apoptotic activity. However, most cancers resist TNF-induced programmed cell death [103,104]. An experiment on mice lacking tumor necrosis factor showed that the promotion step must occur during carcinogenesis. Moreover, tumor necrosis factor activates AP-1 (activator protein-1) and NF-κb. These are molecules whose activity may contribute to the progression of carcinogenesis. Manna et al. [103] demonstrated the arrest of reactive oxygen species production and the counteracting of TNF-induced lipid peroxidation by resveratrol. Therefore, blocking the signaling pathway mediated by tumor necrosis factor likely occurs through the elimination of ROS by tested polyphenol. Reactive oxygen species, in turn, are modulators of NF-κb and AP-1, so suppression of these molecules also occurred. In turn, as reported in another publication, exposure of human HT-29 colon epithelial cells to resveratrol and subsequent TNF-α treatment resulted in fewer ROS and reduced inflammation compared to cells not influenced by the polyphenol. Both are important in the course of carcinogenesis [104].

### 8.8. Problems Related to the Use of Resveratrol

Based on the literature shown in Table 1, resveratrol can exhibit antioxidant activity in different ways and diverse models, both in vivo and in vitro. It can also target various types of cancer in experiments. However, despite many studies supporting the vision of resveratrol usage in chemoprevention, it is essential to note the challenges encountered when introducing this polyphenol widely as a chemopreventive agent.

Resveratrol undergoes rapid metabolism and elimination from the body in humans. After oral ingestion, the polyphenol is absorbed by 75%, but a much smaller portion enters the blood and body cells. Through the metabolism of this compound in the liver and intestine, the bioavailability of trans-resveratrol oscillates between 1–2%. Attempts are being made to use liposomes, micelles, emulsions, polymeric nanoparticles, or nanocrystals to improve the bioavailability and water solubility of the antioxidant. The antiproliferative effects of free resveratrol and resveratrol-loaded lipid-core-nanocapsules were investigated in colon cancer cells. The study’s findings clearly showed that cytotoxicity depends on dosage and time. Furthermore, resveratrol has been shown to cause cancer cell death via the apoptotic pathway. Moreover, resveratrol was shown to have a significant apoptotic impact. Free resveratrol produced almost 15% of cell death. Still, the resveratrol-loaded lipid-core-nanocapsule generated a significant 36% cell apoptosis, indicating a more significant anticancer effect in cancer cells [105]. Another option is natural or synthetic derivatives of resveratrol characterized by better biological activity, lower toxicity, and fewer side effects [106]. Although 3,5,4′-trihydroxystilbene is considered a safe substance, there are cases of reported adverse sequelae of administration of this antioxidant and the occurrence of toxicity. Both the dose of resveratrol and the state of the environment relative to the redox equilibrium in which the administered substance will be found affect whether the effect will be beneficial [107].

Another study used liposomes to encapsulate pristine resveratrol with cyclodextrin–resveratrol capping complexes in lipophilic and hydrophilic compartments to develop and optimize a new drug carrier. It was demonstrated that the new nanopreparations released the drug at 100% compared to free resveratrol and traditional liposomal preparations, which had a 40–60% drug release profile. The cytotoxicity of resveratrol-encapsulated liposomes on colon cancer cell lines was also investigated. Compared to free resveratrol, the cytotoxicity profile of liposomes is dose-dependent and reported to be superior [108]. Nanocapsules with a lipid core loaded with trans-resveratrol were also tested and found to reduce the viability of glioma cells to a greater extent than resveratrol (in vitro study). Trans-resveratrol-loaded lipid core nanocapsules significantly reduced glioma cell viability. Nanocapsules reduced glioma cell viability by inducing apoptotic cell death. These findings suggest that nanoencapsulation of resveratrol increases its effect on glioma, and the problems related to its low bioavailability can be solved using new technologies [109].

**Table 1:** **Resveratrol in the chemoprevention of different cancers.** The table presents various studies describing the importance of resveratrol in chemoprevention, considering the specific mechanism, carcinogen, and type of cancer.

Research Model	Carcinogenic Factor	Effect Exerted by Resveratrol	Target of Chemoprevention	Author
In vivo	Benzo[a]pyrene	Suppression of CYP1A1 expression, inhibition of DNA adduct formation	Lung cancer	[76]
In vitro	TCDD	Inhibition of CYP1B1 activity and expression, prevention of DNA damage	Breast cancer	[83]
In vitro	tBHP, Fe^2+^	Alleviation of oxidative stress	Pheochromocytoma	[84]
In vivo	DENA	Alleviation of oxidative	Liver cancer	[85]
		stress		
In vitro	–	Elimination of the tyrosyl radical	Leukemia	[88]
In vitro	–	Modulation of iNOS and reduction of NO synthesis	Gastric cancer	[89]
In vitro	LPS	Downregulation of iNOS expression and inhibition of NO synthesis	Colon cancer	[90]
In vitro	UVB	Reduction of oxidative stress, inhibition of NF-κb	Skin cancer	[110]
In vitro	TNF	Reduction of ROS production and lipid peroxidation, suppression of AP-1 and NF-κb	Lymphoma, cervical cancer, glioma, leukemia	[103]

## 9. Curcumin

The second chemopreventive compound with antioxidant properties discussed in this paper is curcumin, diferuloylmethane. This substance can be found in the rhizome of turmeric (Curcuma longa), which accounts for 5 to 10% of its dry weight. This plant is most commonly found in East Asia and India. Like resveratrol, the compound is included in the polyphenol group [111]. The curcumin contained in turmeric is among the ingredients in the curry spice used worldwide, and it is responsible for its characteristic yellow-orange color. However, the genuine interest stems from its health properties [58,111]. The potential use of curcumin in chemoprevention methods and as a chemotherapeutic agent is being explored. This polyphenol has shown anti-inflammatory and anticancer properties, as well as aiding in treating viral and bacterial infections [111]. Curcumin also has the potential to alleviate respiratory and liver diseases or diabetic wounds [112]. Associated with the properties of this compound is the presence of hydroxyl and methoxyl groups in its molecule [113].

### 9.1. Antioxidant Properties of Curcumin

Curcumin can be found in two tautomeric forms: enol or ketone. The former is more stable and outperforms the ketone form in its antioxidant activity. Curcumin’s chelation of metal ions has been observed to enhance its antioxidant properties.

**Figure 5:**
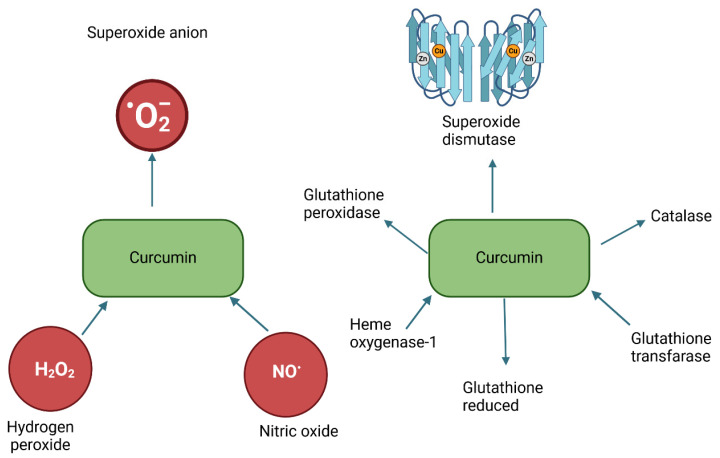
**Curcumin’s antioxidant properties.** (**Left**) Curcumin can react directly with ROS. (**Right**) Curcumin upregulates many antioxidant defense system components such as GPx, CAT, GST, SOD, GSH, and heme oxygenase-1 [118].

Excessive amounts of Mn^2+^, Zn^2+^, Cu^2+^, and Fe^2+^ in the brain contribute to neurodegenerative diseases by linking to processes such as protein aggregation and oxidative stress, so the use of curcumin, which is both a metal ion chelator and antioxidant, may be effective in this case [114]. Curcumin effectively chelates iron ions involved in the Fenton reaction, the product of which is a hydroxyl radical. When free Fe^2+^ decreases, no •OH is formed, and oxidative damage is reduced [115]. The occurrence of phenolic groups in the structure of diferuloylmethane, which enables it to react with ROS and RNS, may cause its antioxidant activity. The chemopreventive properties of curcumin related to its antioxidant nature involve the regulation of enzymes affecting redox balance in the body and the detoxification of carcinogens (superoxide dismutase, catalase, glutathione S-transferase, heme oxygenase, NAD(P)H: quinone reductase, glutathione peroxidase, and glutathione reductase) [116]. In addition, chemopreventive effects include eliminating reactive oxygen species or regulating nitric oxide production [115]. This polyphenol eliminates reactive oxygen and nitrogen species: •OH, 1O2, NO, O •−, or H O. Like resveratrol, curcumin protects DNA and lipids from oxidation [117] (Figure 5).

### 9.2. The Role of Curcumin in the Detoxification of Carcinogens

As demonstrated by Garg et al. [119], mice on a diet containing curcumin, later exposed to benzo[a]pyrene, had higher levels of glutathione S-transferase or NAD(P)H: quinone reductase, along with increased expression of their mRNA and activity in organs such as the liver and brain, than animals fed a standard diet also exposed to the carcinogen. The number of GST isomers in the liver and lungs also increased. Parallel to these changes, activation of Nrf2 was observed. Together, these processes resulted in better benzo[a]pyrene detoxification. Moreover, the levels, expression, and activity of enzymes induced by the action of the applied carcinogen, such as CYP1A1 and 1A2, in the lungs and liver decreased. By modulating phase I and phase II enzymes, curcumin reduced the formation of DNA adducts, oxidative damage, and inflammation. In a study on MCF-7 human breast cancer cells, curcumin inhibited CYP1A1 enzyme activity induced by 7,12-dimethylbenz[a]anthracene [111]. This antioxidant also presented chemopreventive properties in the case of oxidative stress induced in chicken liver by aflatoxin B1 (AFB1), which has carcinogenic, mutagenic, immuno-, and hepatotoxic effects. Hepatic enzymes of the CYP450 family are responsible for the bioactivation of this harmful compound, while phase II enzymes participate in detoxification under Nrf2 regulation. The presence of AFB1 in the diet decreased the activity of antioxidant enzymes in the liver and blocked nuclear transcription factor and phase II enzymes while increasing the level of CYP450 enzymes. Curcumin administration had the opposite effect. The activity and expression of glutathione S-transferase, which is crucial in the detoxification process of aflatoxin, and glutathione peroxidase increased.

In addition, the level of Nrf2 was raised, while CYP3A4, 1A2, and 1A1 expressions were reduced [120]. Thapliyal and Maru [121] showed that the inhibition of CYP2B1 activity induced by benzo[a]pyrene, CYP1A2, and CYP1A1 initiated with phenobarbital occurs under the influence of curcumin. Choi et al. [122] reported that diferuloylmethane inhibited CYP1A1 and 1B1 activity under the influence of 2,3,7,8-tetrachlorodibenzo-p-dioxin. Moreover, there was no neoplastic transformation of normal cells treated with curcumin. In another experiment, phase II enzyme activities were examined in mice receiving said antioxidant and food. Glutathione S-transferase and quinone reductase activities were increased in the liver and kidney. The obtained results concluded that the studied polyphenol possesses cell-protective properties against carcinogenesis by enhancing the detoxification of carcinogens in the body [123]. In a study using mice with Dalton’s lymphoma, Nrf2 activity increased in animals receiving curcumin. Consequently, levels comparable to control phase II detoxification enzymes were also restored (GST, GR, and NAD(P)H: quinone reductase). In addition, the antioxidant affected inflammation by regulating iNOS and COX-2, which show higher expression in tumor-bearing animals than in healthy individuals and contribute to nitric oxide generation, inflammation, and tumor progression [124].

### 9.3. Curcumin Counteracts the Oxidation of Lipids, Proteins, and DNA

Curcumin prevents LDL peroxidation induced by reactive oxygen species. It has been postulated that the presence of a phenolic group in the diferuloylmethane molecule is a necessary factor for such activity since derivatives lacking it do not stop oxidation induced with cupric ions or AAPH—2,2′-azobis(2-amidinopropane hydrochloride) [64]. Since low-density lipoprotein oxidation and increased oxLDL levels have been positively correlated with various cancers, they too may become targets for chemopreventive agents [92]. Naidu and Thippeswamy [125] concluded that curcumin significantly reduces LDL oxidation initiated by incubating lipoproteins with CuSO_4_. Wei et al.[126] demonstrated the inhibition of lipid peroxidation and protein oxidation by curcumin in rat liver mitochondria. Oxidative stress was induced with AAPH to produce alkyl radicals, and iron ions combined with vitamin C to produce hydroxyl radicals. A similar effect was obtained by Okada et al. [127] in a study of the degree of DNA damage and lipid oxidation caused by ferric nitrilotriacetate (Fe-NTA)-induced oxidative stress, often leading to kidney cancer in rodents. Both parameters were reduced under the influence of curcumin. Administration of the same compound to mice in which Ehrlich solid tumor was induced raised the levels of superoxide dismutase and catalase in various tissues. In addition, the concentration of malondialdehyde (MDA), which serves as a marker of lipid peroxidation, was checked. Tumor growth increased levels of this compound in the kidneys, testes, and liver.

On the other hand, the effect of curcumin caused it to decrease in the liver, meaning inhibition of lipid peroxidation [128]. Ak and Gülçin [129] also concluded that diferuloylmethane’s high affinity for iron ions and their chelation may be the primary mechanism for inhibiting lipid peroxidation. Administration of curcumin to residents of West Bengal, India, exposed to groundwater contaminated with the carcinogen arsenic for three months minimized the amount of reactive oxygen species formed and concomitant DNA damage and lipid peroxidation in lymphocytes compared to those who did not receive such supplementation. Exposure to arsenic has been linked to skin, lung, bladder, liver, or kidney cancer, but the disease can occur even after 20 years [130]. Diferuloylmethane showed a protective effect on human lymphocytes taken from volunteers and incubated with radioactive iodine 131 (^131^I) by preventing genotoxicity. This is evidenced by a reduction in the incidence of micronuclei, which indicates chromosome damage. Radiation induced by isotope administration results in oxidative stress, suggesting a role for curcumin as a free radical scavenger in protecting lymphocytes from genotoxic effects. This is a noteworthy fact since radioactive iodine therapy for the treatment of thyroid cancer can cause secondary cancers, and the use of agents that protect the body from damage to genetic material reduces the risk of secondary cancer [131]. In another experiment, the authors focused on the effect of diferuloylmethane on the following oral conditions: leukoplakia, submucosal fibrosis, and lichen planus. The mentioned lesions appearing in this area of the body can become a substrate for developing oral cancer. The main risk factors for this type of cancer include chewing tobacco, smoking, and drinking alcohol. After a week’s intake of polyphenols in the saliva and serum of the subjects in all three categories considered, there was an increase in the levels of vitamins C and E, which have antioxidant properties, and a decrease in malondialdehyde (a marker of lipid peroxidation) and 8-OHdG (8-hydroxy-2′-deoxyguanosine, a marker of DNA damage). The results suggest that curcumin can be used in oral cancer chemoprevention, as it alleviates precancerous lesions by reducing oxidative stress [132].

### 9.4. Curcumin Has Inhibitory Effects on NF-κB, AP-1, and Related Molecules

Curcumin affects the NF-κB pathway, decreasing this transcription factor’s expression. It stimulates interleukins, iNOS, TNF-α, and COX-2 in inflammatory processes. NF-κB is considered one of the most critical elements involved in cancer development. In the context of carcinogenesis, reactive oxygen species can cause activation of iNOS, NF-κB, TNF-α, and COX-2. Oxidative stress accompanies many types of cancer and the processes leading to their emergence. Activated nitric oxide synthase generates NO, and cyclooxygenase-2 indirectly contributes to forming reactive oxygen and nitrogen species. In addition, findings support the involvement of NO in increasing the risk of mutation of the suppressor gene p53, whose alterations are important for the development of malignant tumors. Curcumin reduces TNF-α expression, counteracts inflammation, and inhibits TNF-dependent NF-κB pathway activation induced by H_2_O_2_ or phorbol esters [133,134]. As one possible mechanism for this phenomenon, the authors of a study pointed to the antioxidant’s reduction of reactive oxygen species production, as an increase in their levels is observed in response to TNF, in addition to ultraviolet radiation, lipopolysaccharide, or interleukin 1 [135].

Curcumin additionally arrests AP-1 activity induced by cell exposure to promoters of carcinogenesis [136]. It is known that reactive oxygen species can activate transcription of this molecule, while inhibition of the process counteracts the neoplastic transformation of JB6 cells and human keratinocytes [116,135]. Elevated levels of AP-1 expression are required for carcinogenesis promotion and progression stages. Given the link between ROS, AP-1 activity, and carcinogenesis progression, curcumin’s behavior as a free radical scavenger is a mechanism with potential application in chemoprevention. In addition, this polyphenol can downregulate TNF-α-induced AP-1 expression [135].

In one study that proved the link between AP-1 and NF-κB expression levels and the effects of curcumin, human lung adenocarcinoma cells of the A549 line were used. Following treatment with hydrogen peroxide and TNF-α, a significant increase in NF-κB, AP-1 activity, and interleukin 8 (IL-8) release was observed. All three types of molecules are associated with inflammation. Curcumin administration caused the opposite phenomenon and a halt in NF-κB and AP-1 activity, as well as H_2_O_2_-induced IL-8 and TNF-α release. In addition, an increase in the expression of reduced glutathione (GSH) and GCLC occurred. Also, a direct reaction of diferuloylmethane with O •− and •OH occurred. The regulation of NF-κB and AP-1 activity and the elimination of free radicals attest to the antioxidant properties of curcumin, as well as its anti-inflammatory effects, which can be used in chemoprevention [119].

It has also been shown that a decrease in NF-κB and the enzymes it regulates can occur under the influence of curcumin. Prakobwong et al. [137], in an experiment conducted on hamsters given N-nitrosodimethylamine (NDMA) and infected with the Opisthorchis viverrini fluke, induced the animals to develop cholangiocarcinoma. In Southeast Asia, exposure to NMDA contained in food and infections with the parasite above species cause the occurrence of this dangerous and metastatic cancer. Curcumin has been shown to inhibit the expression of iNOS and COX-2, as well as NF-κB, which regulates these two enzymes, reducing inflammation and reducing DNA damage caused by reactive oxygen and nitrogen species. The same authors also presented a result suggesting that in hamsters infected with O. viverrini, curcumin reduced oxidative and nitrosative DNA damage by equalizing the redox state. Suppression of genes whose activity causes deoxyribonucleic acid destruction (genes encoding NF-κB, COX-2, and iNOS) and an enhancement of the expression of those causing the opposite effect, that is, antioxidant (genes encoding CAT, SOD2, and SOD3) effects occurred. This demonstrates the potential of curcumin as a chemopreventive agent against cholangiocarcinoma [138].

### 9.5. Curcumin Increases Heme Oxygenase Expression and Activity

Curcumin regulates the expression of heme oxygenase (HO-1) by activating various kinases. HO-1 is an enzyme with antioxidant and anti-inflammatory properties involved in the breakdown of heme. An experiment on human monocytes and macrophages treated with lipopolysaccharide showed that diferuloylmethane induces HO-1 expression and activity [139]. Another study suggested that the induction of heme oxygenase by curcumin may partially inhibit iNOS, COX-2, and 5-LOX activity, the overexpression of which contributes to oxidative and nitrosative stress [140]. Heme oxygenase is an enzyme regulated by the transcription factor Nrf2; the expression of the gene encoding HO-1 depends on this protein. In one study, in which curcumin was administered to hens and its effect on ovarian cancer development was subsequently tested, HO-1 and Nrf2 levels were increased [141]. The polyphenol also counteracts oxidative damage caused by ionizing radiation by increasing the expression of HO-1 and γ-GCS (γ-glutamyl cysteine synthetase) in rats’ brains [142].

### 9.6. The Effect of Curcumin in Different Research Models and Problems Associated with Its Use as a Chemopreventive Agent

Although curcumin’s chemopreventive and anti-inflammatory effects have been confirmed in numerous in vitro and in vivo studies, its use has certain limitations. This is also the reason for not introducing it to the pharmaceutical market. The reason is the pharmacokinetic limitation of curcumin. Appropriate absorption, distribution, metabolism, and excretion rates ensure that drugs act on the human body and fulfill a therapeutic role. Low water solubility, poor absorption and elimination, and rapid metabolism limit the use of curcumin. The water solubility of curcumin is inadequate, and its physical and chemical properties are unstable. Only a small amount of curcumin dissolves in the digestive tract, and effective ingredients are limited. Curcumin is a robust hydrophobic compound with approximately 11 ng/mL solubility. Curcumin is extremely unstable in alkaline environments, forming degradation and autoxidation products such as ferulic acid, feruloylmethane, vanillin, and bicyclopentadione [143,144,145].

Curcumin is very poorly absorbed from the gastrointestinal tract. The maximum safe oral dose of curcumin is 12 g/day, but the final serum curcumin level is only about 50 ng/mL [144] This may be due to the action of P-glycoprotein and the first-pass effect through the liver, which is caused by curcumin being a substrate for P-glycoprotein, a transmembrane, ATP-dependent drug efflux pump that excretes curcumin from the intestinal membrane, thereby limiting its permeability [146].

The hepatic first-pass effect causes some curcumin to be metabolized in the intestinal mucosa and the liver, which makes it less absorbable [147].

The poor bioavailability of curcumin is caused by its rapid metabolism. After entering the blood, curcumin is quickly metabolized as an inactive substance. After oral administration of curcumin, most of it is excreted along with its metabolites. Only a small amount enters the bloodstream to be used, among others, as a chemopreventive substance. Certain limitations in using curcumin in chemoprevention prompted searching and creating analogs with better effects [112]. For example, a study on a mouse model of colon cancer found that the synthetic compound GO-Y030 has a more effective chemopreventive impact than the original substance [148]. Curcumin is characterized by low toxicity but also low bioavailability after oral administration. It is rapidly metabolized, conjugated in the liver, and excreted in the feces. After administering 40 mg/kg of this polyphenol to rats, it is completely removed from the plasma after about an hour. As with resveratrol, various ways of delivering the substance are being attempted to solve the problem. Liposomes, nanoparticles, micelles, or phospholipid complexes may be used. Another option is to create analogs with greater bioavailability, such as PACs [112,149]. Examples of recent relevent use of curcumin as an antioxidant are presented in Table 2.

## 10. Quercetin

Quercetin (3, 3′, 4′, 5, 7-pentahydroxyflavone) is a polyphenol present abundantly in the human diet, as it can be found in a wide variety of fruits and vegetables, with the most significant amount in red onions and shallots [150]. Broccoli, kale, tomatoes, apples, red grapes, cherries, berries, tea, and red wine are also rich sources of quercetin [151]. This compound can be used to dye cotton or as a chemical analysis reagent that chelates metal ions. When administered with calcium salts, it exhibits antiallergic properties that arise from inhibiting the production and release of allergic reaction mediators, such as histamine. Quercetin shows potential for preventing pulmonary and cardiovascular diseases, osteoporosis, and cancer. Antiviral and antibacterial effects also characterize it. It can be helpful in the treatment of diabetes, and its consumption reduces the risk of cataracts and improves vision. Much of quercetin’s observed effects on the body come from its antioxidant properties [150,152]. The 3,3′,4′,5,7-pentahydroxyflavone molecule contains five hydroxyl groups and three aromatic rings and is characterized by a yellow color in crystalline form. In food, this polyphenol is often bound to phenolic acids, alcohols, or sugars, and its average intake ranges between 25 and 35 mg per day [150]. Additionally, it can be found in free form in hawthorn or chestnut [152]. Sources and uses of quercetin are presented in Figure 5.

### 10.1. Antioxidant Properties of Quercetin

**Figure 6:**
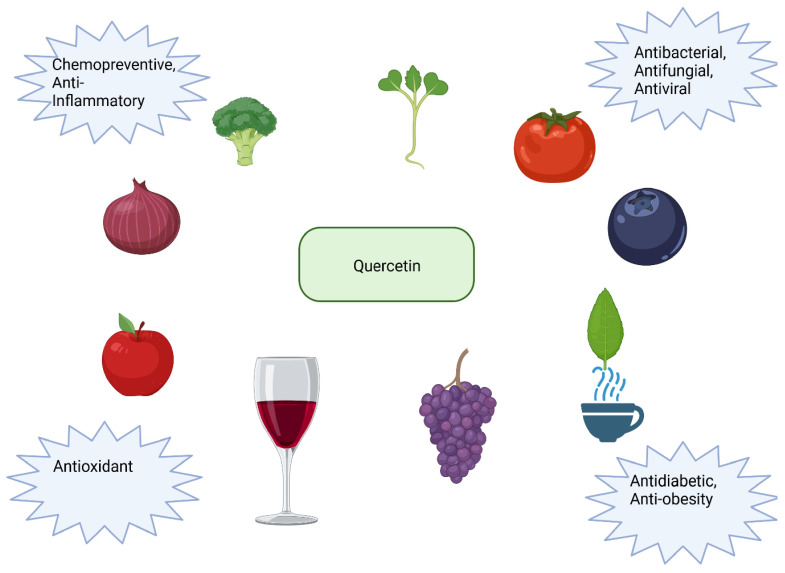
**Sources and uses of quercetin.** Quercetin is present in the human diet, as it can be found in a wide variety of fruits and vegetables, with the most significant amount in red onions and shallots, broccoli, kale, tomatoes, apples, red grapes, cherries, berries, tea, and red wine. Quercetin is an antibacterial/viral/fungal, anti-inflammatory, antioxidant, and anti-obesity and is also chemopreventive [150,151,152].

**Figure 7:**
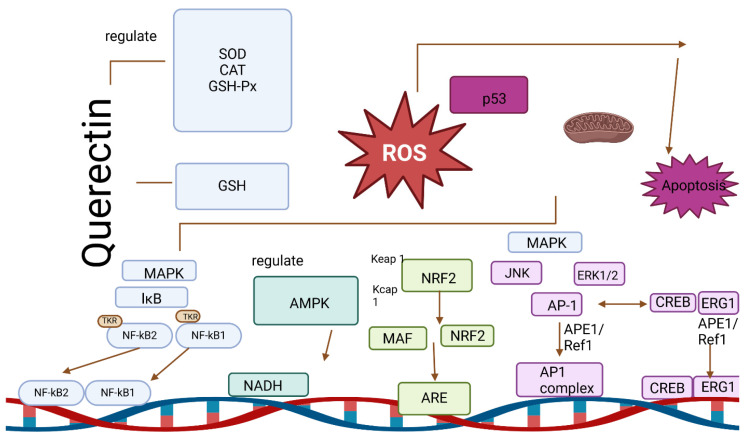
**The antioxidant signaling pathway is regulated by quercetin.** Environmental factors increase the production of ROS. The mitochondrial electron transport chain is another robust source of intracellular ROS generation. Quercetin can regulate the enzyme-mediated antioxidant defense system and the non-enzyme-dependent antioxidant defense system. It can also restrict signal pathways such as NRFB, AMPK, and MAPK caused by ROS to promote the antioxidant defense system and maintain oxidative balance. ROS, in turn, enhance the production of APE1/Ref1 and the activation of several signaling events, including p53-mediated apoptotic events, MAPK pathways, the NF-E2-related factor (NRF2)-mediated activation of genes containing antioxidant response element (ARE), and NF-κB [67,154,155].

As a result of quercetin’s maintenance of the oxidoreductive balance, cells remain protected from oxidative stress. A study dedicated to determining the relationship between the risk of various chronic diseases and flavonoid consumption showed a lower risk of lung cancer in a group of men supplementing higher doses of quercetin. The preventive effect of the antioxidant was attributed to its antioxidant properties [153]. Quercetin’s antioxidant activity primarily manifests by its impact on enzymatic activity, ROS, and signaling pathways that external factors have induced. In a study conducted on rats, a manganese-induced decrease in antioxidant enzymes and an increase in oxidative acetylcholinesterase, hydrogen peroxide, and the degree of lipid peroxidation were performed. Administration of quercetin alleviated all the adverse changes mentioned and prevented manganese poisoning in animals. The described antioxidant regulates the levels of glutathione, heme oxygenase-1, and superoxide dismutase, increasing the antioxidant capacity of the organism. It also prevents cholesterol oxidation, which counteracts the development of atherosclerosis and vascular and heart diseases [152]. In vitro studies have noted that quercetin metabolites from the liver and enterocytes act as antioxidants by inhibiting the oxidation of LDL cholesterol, which contributes to atherosclerotic plaque formation [151]. By affecting various signaling pathways, quercetin can improve the antioxidant capacity of cells. This occurs due to the regulation of ROS-induced pathways, with subsequent modulation of Nrf2, NF-κB, or AP-1 activity [67]. In addition, quercetin can affect the levels of antioxidant enzymes that regulate the activity of the mentioned molecules. For example, it has been shown that the induction of HO-1 by quercetin can inhibit the activity of AP-1 [154]. In addition, as an inhibitor of nitric oxide synthase, it counteracts the formation of RNS and inhibits the activity of the oxidative enzyme xanthine oxidase [155]. The antioxidant signaling pathway regulated by quercetin is presented in Figure 6 and Figure 7.

### 10.2. Effects of Quercetin on Carcinogen Metabolism

Like the previously described antioxidants, quercetin affects various enzymes metabolizing carcinogens. 3,3′,4′,5,7-pentahydroxyflavone decreases the activity of phase I enzymes such as CYP1A2 and CYP3A4 and increases the activity of the following phase II enzymes: glutathione S-transferase and quinone reductase [156]. Quercetin also reduced the activity of CYP1A1 and 1B1 and reduced oxidative stress in mice treated with 7,12-dimethylbenz[a]anthracene, a carcinogenic compound [157]. Kim et al. reported the results of a study conducted on rats and the human liver cancer cell line HepG2, in which quercetin alleviated benzo[a]pyrene-induced toxicity. Metabolization of this carcinogen in the liver leads to the production of reactive oxygen species and the formation of oxidative DNA damage [158]. In addition, one of its metabolites, benzo[a]pyrene-7,8-dihydrodiol-9,10-epoxide (BPDE), tends to combine with DNA to form adducts. Both types of damage described can induce distortion of the double helix, leading to cancer development. Oral administration of quercetin to rats for 30 days reduced the formation of oxidative DNA damage and DNA adducts in the liver and stomach, while there was a reduction in the level of reactive oxygen species in the cell line. The chemopreventive effect of the antioxidant involved stimulation of detoxification of the carcinogen and its metabolites, as modulation of glutathione S-transferase levels occurred. The impact of quercetin on the regulation of CYP1A1 and CYP1B2 was also demonstrated. In another study, the hepatic activity of the detoxifying enzymes glutathione peroxidase and glutathione S-transferase increased in rats administered quercetin contained in water, with the latter enzyme elevated significantly [159]. Quercetin has also been shown to increase the activity of NAD(P)H: quinone reductase in MCF-7 human breast cancer cells, which could be a way to protect against carcinogens [160].

### 10.3. Quercetin Alleviates Oxidative Damage to DNA

DNA damage causes genomic instability and genetic mutations, making it an important factor in carcinogenesis and a valid aspect of chemoprevention strategies. Quercetin protects DNA due to its antioxidant properties, which can be used to prevent colon, lung, or liver cancer. DNA damage and ROS concentrations are reduced by quercetin’s regulation of antioxidant enzyme levels. In one experiment, carcinogenesis was induced by 1,2-dimethylhydrazine (DMH) in the colon tissue of rats. The study results showed that quercetin causes a significant reduction in oxidative DNA damage and induces DNA repair through the Nrf2 pathway. In addition, a lower incidence of colon tumors was observed in polyphenol-treated animals [161]. In another experiment, quercetin supplementation in rodents exposed to benzo[a]pyrene prevented the development of lung cancer due to mitigating oxidative damage to DNA [162]. 3, 3′, 4′, 5, 7-pentahydroxyflavone has also shown a chemopreventive effect on liver cancer induced by N-nitroso diethylamine (NDEA) in rats. This phenomenon occurred through a significant reduction in oxidative stress by quercetin, thereby reducing oxidative DNA damage and lipid peroxidation. In the experiment, the effect of NDEA on the genetic material of liver cells resulted in the appearance of mutations in the p53 gene associated with carcinogenesis. At the same time, no such changes were found when quercetin and NDEA were used simultaneously [163]. In the human colorectal adenocarcinoma cell line Caco-2, quercetin inhibited hydrogen peroxide-induced oxidative DNA damage [164]. In a similar study using H_2_O_2_ and the same cell line, quercetin reduced oxidative damage to colon cells and levels of reactive oxygen species. In addition, increased activity of GCLC and GSH has been observed. More reactive oxygen species are produced in the colon than in the small intestine. During oxidative stress, the enzymes present in this organ do not effectively reduce oxidative DNA damage, so the use of antioxidants to restore redox balance and eliminate inflammation may prove to be a helpful strategy for diseases such as IBD (inflammatory bowel disease) and colon cancer, where abnormal H_2_O_2_ regulation has been positively correlated with its occurrence [165]. The antioxidant properties of quercetin protect cells from the process of carcinogenesis by reducing oxidative DNA damage, but this happens not only by eliminating ROS and RNS. According to one publication, DNA protection against reactive oxygen species also occurs indirectly through increased protein expression that limits free radical DNA damage, for example, p53 [166].

### 10.4. Elimination of Reactive Oxygen and Nitrogen Species by Quercetin

Quercetin may exhibit a chemopreventive effect by capturing and eliminating reactive nitrogen and oxygen species. The protective effect of this antioxidant was demonstrated on HaCaT keratinocytes exposed to UVB radiation by removing ROS. Decreased levels of reactive oxygen species prevented cellular damage, such as lipid peroxidation and mitochondrial damage [167]. In another study conducted on hamsters in which the development of buccal pouch carcinomas was induced with 7,12-dimethylbenz[a]anthracene, the generation of ROS was inhibited under the influence of quercetin. Consequently, no activation of NF-κB can contribute to carcinogenesis [168]. In addition, quercetin can induce apoptosis of tumor cells in Dalton’s lymphoma-bearing mice by reducing ROS levels. However, the ability of polyphenols to induce tumor cell apoptosis is often attributed to their pro-oxidant properties through a mechanism that leads to increased production of reactive oxygen species.

Furthermore, a reduction in protein kinase C (PKC) activity, which is subject to partial control by reactive oxygen species, was observed after quercetin administration to animals. Therefore, it was suggested that the reduction of ROS occurs through a mechanism involving PKC. Protein kinase C is a molecule that plays an essential role in regulatory processes, but under oxidative stress, it exhibits effects promoting carcinogenesis and may contribute to the progression stage. In addition, PKC activation is a required factor for the production of NADPH oxidase-dependent reactive oxygen species. The experiment’s results indicate that quercetin has chemopreventive effects against Dalton’s lymphoma in mice by initiating apoptosis and regulating PKC signaling as a consequence of eliminating reactive oxygen species and alleviating oxidative stress [169]. In a study on mice exposed to gamma radiation, quercetin reduced the production of hydroxyl radicals and superoxide anion radicals [170]. This compound also reduces elevated nitric oxide and reactive oxygen species levels in the lipopolysaccharide-treated human acute monocytic leukemia cell line THP-1 [171]. Wadsworth and Koop [172] reported that quercetin eliminated nitric oxide, an excess of which accompanies chronic inflammation in macrophages exposed to lipopolysaccharides.

### 10.5. Effect of Quercetin on Nitric Oxide Synthesis

Quercetin can inhibit carcinogenesis by affecting nitric oxide synthase and NO. While nitric oxide has been shown in some studies to have an anticancer effect, it has also been proven to be associated with processes such as DNA damage formation, apoptosis, activation of oncogenes, and inhibition of suppressor genes and DNA repair enzymes activity, simultaneously indicating its role in carcinogenesis. In addition, iNOS expression is observed in various types of cancer (lung, breast, bladder, prostate, colorectal cancer, melanoma) and is associated with poor survival in cancer patients [173,174]. In lipopolysaccharide-treated BV2 mouse microglia cells, quercetin reduced nitrosative stress by decreasing the expression of both iNOS and NO with the involvement of the transcription factor kappa B and by inducing Nrf2-mediated heme oxygenase expression [175]. In a study by Raso et al. [176], quercetin stopped NO synthesis through modulation of iNOS in J744A.1 macrophage, while Liang et al. [177] found that based on the results of an experiment in which quercetin inhibited nitric oxide synthase expression in mouse macrophages, there is a potential significance of this phenomenon in inflammation and carcinogenesis.

### 10.6. Quercetin Counteracts Lipid Peroxidation and LDL Oxidation

Quercetin benefits human health and has a chemopreventive impact due to reduced lipid peroxidation and LDL oxidation. This polyphenol integrates into the structure of the lipid bilayer and eliminates emerging free radicals such as •OH, which prevents the oxidation of lipids that make up the cell membrane [178]. In one study, quercetin significantly reduced lipid peroxidation in a rat model of prostate cancer induced by testosterone and the carcinogenic N-methyl-N-nitrosourea (MNU) [179]. The same result was obtained when the antioxidant was administered two hours before the induction of hepatocellular carcinoma in rats and their exposure to diethylnitrosamine [180]. MDA, a marker of lipid peroxidation, was also shown to decrease in human endometrial adenocarcinoma cells after the simultaneous use of quercetin and selenium [181].

Quercetin also counteracts low-density lipoprotein oxidation. The reduction of oxLDL levels by 3, 3′, 4′, 5, and 7-pentahydroxyflavone has been demonstrated both in vitro and in vivo, which may be a potential way to use this antioxidant in chemoprevention [182,183]. Quercetin and its metabolites have also been shown to counteract copper ion-induced LDL oxidation [184]. Moreover, the polyphenol prevented oxidative modification of low-density lipoproteins isolated from human plasma treated with peroxynitrite [185]. In another study, quercetin showed an inhibitory effect on lipoxygenase-induced LDL oxidation [186].

### 10.7. Effect of Quercetin on COX-2, NF-κB, and Nrf2

Results from a study conducted on the HCA-7 colon cancer cell line showed that quercetin decreases cyclooxygenase-2 expression [187]. In a mouse model of benzo[a]pyrene-induced lung carcinogenesis, an increase in COX-2 activity was observed. At the same time, regular administration of quercetin to animals restored this value to the normal range [188]. Quercetin also alleviates oxidative stress and modulates redox signaling induced by ochratoxin A, a mycotoxin with carcinogenic effects, in the human liver cancer cell line HepG2. An increase in Nrf2 expression occurred, while NF-κB and COX-2 expression decreased, preventing DNA damage. The nuclear factor kappa B is subject to activation by reactive oxygen species, followed by the induction of COX-2. Nrf2, on the other hand, is regulated by various antioxidants in response to oxidative stress, and its activation leads to the production of antioxidant enzymes [189]. In an experiment using the same cell line, quercetin suppressed the intracellular production of reactive oxygen species and COX-2 activity induced by tumor necrosis factor. The antioxidant appeared to function as an inhibitor of the NF-κB pathway, which can also undergo TNF-mediated activation [190].

In a study performed on broiler chickens, quercetin was shown to alleviate lipopolysaccharide-induced oxidative stress in the intestines via Nrf2 activation [191]. Also, in an experiment exploring the effects of quercetin and its metabolites on ethanol-treated hepatocytes, Nrf2 activation and subsequent induction of heme oxygenase with antioxidant properties occurred [192].

### 10.8. Quercetin in Chemoprevention and Problems Related to the Use of Quercetin

Like resveratrol and curcumin, quercetin has also shown broad in vitro and in vivo antioxidant activity in models of various types of cancer, as presented in Table 3 based on the literature studies. Nevertheless, it should be noted that quercetin itself possesses certain disadvantageous features that could become an obstacle to creating chemopreventive agents. Quercetin shows low solubility and absorption after oral administration, as with curcumin and resveratrol, and publications regarding improving both properties have begun to appear. In the body, it is metabolized by the small intestine, kidneys, colon, and liver, and its plasma concentration can be reported in units such as nanomoles or micromoles. Higher values are achieved with supplementation, while lower values are achieved with a standard diet. Quercetin phytosomes, a formulation based on food-grade lecithin, administered to healthy volunteers, resulted in increased absorption and solubility compared to quercetin alone. They also proved to be tolerated and caused no adverse effects [163,193]. The bioavailability of quercetin can be improved using various delivery systems, such as microparticles, encapsulation, phospholipid complexes, or hydrogels. When administered this way, the compound often exhibits more potent antioxidant properties than quercetin [194] that naturally occurs in plants. Polyphenol has the best absorption and solubility in the form of glycosides. However, it should be noted that despite the potential obstacles that can be encountered regarding the degree of solubility and absorption of quercetin, its metabolites have a relatively long half-life of 23 to 28 h, so an accumulation in the body may occur. Consumption of quercetin for prolonged periods is inadvisable for people struggling with hypotension and blood clotting disorders [153]. Based on one study regarding the toxicity of quercetin in rats and two other experiments using the same rodent species as a model, it was concluded that high doses of quercetin may exacerbate nephrotoxic effects, especially in cases where previous kidney damage has been present. In humans, however, side effects from supplementation with this antioxidant are rarely observed, and existing reports present the mild nature of the adverse effects described [195]. In addition, in recent years, studies have been conducted on quercetin analogs showing better chemopreventive properties in given cancer models [175,196].

Despite numerous in vitro and in vivo anticancer studies of quercetin nanoformulations, there are still some limitations in their clinical translation, such as cost, safety, and side effects [197]. The in vivo anticancer potential of quercetin nanoparticles has been evaluated in various tumor models, among which oral administration is the most preferred route of quercetin nanoformulations. Although various quercetin nanoparticles capable of increasing the bioavailability of quercetin in the body have been synthesized, the need for more stable and specific nanoparticles remains challenging.

Reducing the side effects and toxicity of quercetin nanoparticles before their clinical use is crucial. Incorporating tumor-cell-specific targeting moieties into the nanoparticles can enhance their targeted delivery and reduce their interaction with normal cells, thereby preventing side effects. Meeting the safety criteria set by government authorities for nanopreparation synthesis is necessary [198]. The subsequent challenge is to ensure the cost-effectiveness of nanopreparations, emphasizing the need for more affordable quercetin-based drugs.

To sum up, in the case of using quercetin in chemoprevention, we have problems similar to those of the other substances discussed. The first is related to the bioavailability and absorption of the substance, and the second is associated with the need to conduct numerous clinical trials and adapt to the legislation of a given country regarding the quality and safety standards of a given medicinal product. The next challenge concerns the economic sphere so investors can make money on the drug containing quercetin and production costs are profitable for pharmaceutical companies.

## 11. Resveratrol, Curcumin, and Quercetin Targeting the Thioredoxin System

Thioredoxin (Trx), NADPH, and thioredoxin reductase (TrxR) form the thioredoxin system found in almost all living cells. It functions in thiol-dependent thiol-disulfide exchange reactions, which are crucial for controlling reduced intracellular redox balance, cell growth, defense against oxidative stress, and the control of apoptosis. It also plays a multifaceted role in mammalian cells, including cancerogenesis. Reduced Trx activates the binding of transcription factors to DNA and participates in antioxidant defense by repairing oxidatively damaged proteins or as an electron donor for peroxiredoxins. The Trx system synthesizes deoxyribonucleotides for DNA synthesis, replication, and repair by ribonucleotide reductase. Trx and truncated Trx (Trx80) modulate immune cell functions. TrxR isoforms in the cytosol and mitochondria are essential selenoenzymes with selenocysteine in the active site. These enzymes are also targets for existing chemotherapy drugs. Numerous scientific reports describe higher expression of Trx and TrxR in some cancers. Data in the literature suggest that high levels of Trx may be associated with chemotherapy resistance.

In contrast, others suggest that high levels of Trx and TrxR may induce apoptosis and reduce the mitotic index of some cancers associated with p53-dependent cell death. Recent research indicates that TrxR is essential for carcinogenic processes and the invasive phenotype of cancer. Trx and TrxR are interesting chemotherapy targets [199]. There are also reports in the literature about the possible influence of compounds of natural origin on the thioredoxin system. For example, 3-hydroxyl-containing flavonoids, quercetin, and myricetin have been shown to inhibit the growth of A549 cells by inhibiting cellular thioredoxins. This observed effect correlated with increased levels of oxidized thioredoxin and decreased TrxR activity [200,201].

Curcumin, in turn, inhibits the activity of TrxR, leading to the production of ROS in HeLa cancer cells [202]. Javvadi et al. used the potential of curcumin in radiosensitizing squamous cell carcinoma cells. Due to the ability of curcumin to covalently bind to nucleophilic residues in the C-terminal region of TrxR1, curcumin strongly inhibited its function, increased the release of free radicals, and sensitized cells to radiotherapy [201]. Garcia-Rodriguez et al. studied four natural bioactive compounds (curcumin, resveratrol, melatonin, and silibinin). They investigated their role in redox control and their ability to promote apoptosis in androgen-sensitive and insensitive prostate cancer cells. In this study, these four compounds were shown to differentially affect one of the primary intracellular redox regulators, the thioredoxin system. Curcumin and resveratrol exposure promoted Trx1 oxidation and changed its subcellular localization [203].

Furthermore, resveratrol reduced Trx1 levels in PC-3 cells and increased the expression of its inhibitor TXNIP. On the other hand, melatonin and silibinin only acted as cytostatic agents, reducing ROS levels and showing preventive effects against Trx oxidation. This study demonstrates the importance of the TRX system in terms of differences in their mechanisms of action. These bioactive compounds induce various responses, influencing ROS production and redox systems in prostate cancer cells. This suggests that redox-related pathways may play a key role in processes such as differentiation or survival in prostate cancer [203].

A very important detail is that high Trx and TrxR expression levels in cancer cells lead to drug resistance [204]. The study by Ai and colleagues presents the possibility of using curcumin as a potential drug to reduce cellular drug resistance in cancer. They designed 21 ligustrazine–curcumin hybrids (10a-u) and reported that compound 10d inhibited the proliferation of drug-resistant and drug-sensitive lung cancer cells. It has been observed that 10d inhibits the Trx/TrxR system while increasing intracellular ROS accumulation and apoptosis [205]. In another study, Zhou et al. investigated curcumin analogs (1E,4Z,6E)-5-hydroxy-1-(4-hydroxy-3-methoxyphenyl)-7-(5-methylfuran-2-yl)hepta-1,4,6-trien-3- they (2a). They inhibited the growth of cisplatin-resistant A59 lung cancer cells. Moreover, A549/CDDP cells pretreated with 2a show sensitivity to cisplatin, while TrxR activity is suppressed in them. 2a consequently increases the intracellular accumulation of ROS. It reduces the glutathione (GSH) level and the GSH/GSSG ratio, which indicates a shift of the intracellular redox balance to the oxidative state [206,207].

These studies demonstrate outstanding potential for using polyphenols in the treatment or prevention of cancer and for supporting chemotherapy.

## 12. Conclusions

Based on a review of the literature on the antioxidant properties of three compounds—quercetin, curcumin, and resveratrol—and their potential use in chemoprevention, the following can be concluded: 1. Polyphenols act as free radical scavengers, directly eliminating harmful compounds from cells, and are chelators of metal ions, the participation of which can create reactive oxygen species. Resveratrol, quercetin, and curcumin affect, among others, superoxide dismutase, glutathione S-transferase, heme oxygenase, NAD(P)H: quinone reductase, cyclooxygenase-2, inducible nitric oxide synthase, and cytochrome P450 enzymes. 2. The effects of curcumin, quercetin, and resveratrol include reducing lipid peroxidation and low-density lipoprotein oxidation and reducing oxidative DNA damage that can lead to mutations and cancer. 3. These substances do not show much toxicity, and side effects are rarely reported. Thus, the antioxidant properties of the described polyphenols and their safety enable their use as chemopreventive agents. 4. However, there are some difficulties associated with using polyphenols as chemopreventive agents. The ultimate effect of antioxidants depends on their concentration, the length of time cells are exposed to them, and the type of cell, as polyphenols have been shown to have different effects on normal and cancer cells. 5. Moreover, the low bioavailability and solubility of the described polyphenols make it a challenge to obtain blood concentrations high enough to achieve the intended effects after oral administration. 6. An additional advantage is the possible pro-oxidant effect, increasing the concentration of reactive species and the death of abnormal cells. 7. The high complexity of the mechanisms of action of the substances discussed makes it necessary to conduct numerous, diverse studies on many types of cancer, using both in vitro and in vivo models.

## Figures and Tables

**Table 2:** **Curcumin in chemoprevention of different cancers.** The table presents various studies describing the importance of curcumin in chemoprevention, considering the specific mechanism, carcinogen, and type of cancer.

Research Model	Carcinogenic Factor	Effect Exerted by Curcumin	Target of Chemoprevention	Author
In vivo	AFB_1_	Increased expression and activity of GST, GPx, and Nrf2; decreased expression of CYP3A4, CYP1A2, and CYP1A1	Liver cancer	[120]
In vivo	–	Increased levels of Nrf2, GST, GR, and NAD(P)H: quinone reductase; regulation of iNOS and COX-2	Lymphoma	[125]
In vivo	Fe-NTA	Reduction of lipid peroxidation and oxidative DNA damage	Kidney cancer	[128]
In vivo	Arsenic	Reduced oxidative damage to DNA and lipid peroxidation	Cancer of the skin, lung, bladder, liver, kidney	[131]
In vitro	131I	Reduced oxidative stress and genotoxicity	Secondary cancers	[132]
In vivo	–	Reduced oxidative stress	Oral cancer	[133]
In vitro	TNF-α, H_2_O_2_	Inhibition of NF-κb and AP-1 activity; increased expression of GSH and GCLC	Lung cancer	[137]
In vivo	NDMA, *Opisthorchis viverrini*	Inhibition of COX-2, iNOS, and NF-κb expression, reduction in inflammation and oxidative DNA damage	Cholangiocarcinoma	[138]
In vitro	–	Increased levels of HO-1 and Nrf2	Ovarian cancer	[142]

**Table 3:** **Quercetin in chemoprevention of different cancers.** The table presents various studies describing the importance of quercetin in chemoprevention, considering the specific mechanism, carcinogen, and type of cancer.

Research Model	Carcinogenic Factor	Effect Exerted by Quercetin	Target of Chemoprevention	Author
In vivo/in vitro	Benzo[a]pyrene	Reduction of ROS, oxidative DNA damage, and adducts; regulation of GST	Liver cancer	[158]
In vitro	–	Increase in NAD(P)H: quinone reductase activity.	Breast cancer	[160]
In vivo	DMH	Induction of DNA repair through the Nrf2 pathway	Colorectal cancer	[161]
In vivo	Benzo[a]pyrene	Mitigation of oxidative DNA damage; downregulation of COX-2	Lung cancer	[162,188]
In vitro	UVB	Elimination of ROS, prevention of mitochondrial damage, and lipid peroxidation	Skin cancer	[167]
In vivo	–	Reduction of ROS, induction of apoptosis of cancer cells, reduction of PKC activity	Lymphoma	[169]
In vitro	LPS	Reduction of ROS and NO	Leukemia	[172]
In vivo	Testosterone, MNU	Reduction of lipid peroxidation	Prostate cancer	[179]
In vitro	–	Reduction of lipid peroxidation (effect combined with selenium)	Endometrial cancer	[181]
In vitro	TNF [181], ethanol [183]	Inhibition of ROS production, COX-2 activity, NF-κb pathway [181], Nrf2	Liver cancer	[189,192]
		activation, and HO-1 induction [183]		
